# Supervised and Self-Directed Technology-Based Dual-Task Exercise Training Program for Older Adults With a History of Falls: Mixed Methods Feasibility Study

**DOI:** 10.2196/87577

**Published:** 2026-05-18

**Authors:** Prerna Mathur, Afroditi Stathi, Victoria A Goodyear, Taylor Krauss, Helen Thomas, Angela Cooper, Philip Kinghorn, Caroline Miller, Natalie Ives, Magdalena Chechlacz, Daisy Wilson, Laura Magill, Shin-Yi Chiou

**Affiliations:** 1 School of Sport, Exercise and Rehabilitation Sciences, University of Birmingham, Edgbaston, Birmingham, B15 2TT, United Kingdom, 44 01214142555; 2 Institute for Mental Health, University of Birmingham, Birmingham, United Kingdom; 3 Solihull Community Specialist Falls Service, Solihull Hospital, University Hospitals Birmingham NHS Foundation Trust, Solihull, United Kingdom; 4 Health Economics Unit, Department of Applied Health Sciences, University of Birmingham, Birmingham, United Kingdom; 5 Physiotherapy Department, Queen Elizabeth Hospital Birmingham, University Hospitals Birmingham NHS Foundation Trust, Birmingham, United Kingdom; 6 Department of Inflammation and Ageing, University of Birmingham, Birmingham, United Kingdom; 7 Birmingham Clinical Trials Unit, School of Health Sciences, University of Birmingham, Birmingham, United Kingdom; 8 School of Psychology, University of Birmingham, Birmingham, United Kingdom; 9 Centre for Human Brain Health, University of Birmingham, Birmingham, United Kingdom; 10 Healthcare for Older People Department, Queen Elizabeth Hospital Birmingham, Birmingham, United Kingdom; 11 Birmingham Centre for Observational and Prospective Studies (BiCOPS), School of Health Sciences, University of Birmingham, Birmingham, United Kingdom

**Keywords:** dual-task, aging, falls prevention, exercise, cognition, older adults, technology

## Abstract

**Background:**

Older adults who have fallen are at an increased risk of future falls. Training cognitive and physical functions simultaneously, known as dual-task (DT) training, has been shown to improve mobility and reduce fall risks. With appropriate digital tools, such as smartphones and mobile apps, it is possible to deliver DT training in unsupervised, home-based settings, thereby increasing accessibility beyond the clinical environment.

**Objective:**

This study aimed to evaluate the feasibility and acceptability of a technology-based DT training program delivered through a blended model of supervised and self-directed sessions for older adults with a history of falls. Perspectives of health care professionals working in fall-prevention services were also explored.

**Methods:**

A single-arm, nonrandomized feasibility study was conducted with 45 community-dwelling adults aged 65 years or older with a history of falls. Participants were recruited through primary care practices, secondary care fall-prevention services, and community outreach. The 24-week DT program, which integrated balance and strength exercises with cognitive training using a mobile app, was delivered in two phases: (1) for12 weeks, weekly 50-minute physiotherapist-led group classes in the community, and 2 additional 50-minute self-directed sessions at home, and (2) for 12 weeks, 3 weekly 50-minute self-directed sessions at home. Feasibility and acceptability were assessed through recruitment and retention rates, adherence, app usage, and self-reported satisfaction. Qualitative data were obtained from focus groups with 28 participants who completed the program and 16 health care professionals. Quantitative data were analyzed descriptively, and qualitative data were analyzed thematically.

**Results:**

We recruited 45 of the target 50 participants, with most participants (n=41) recruited through community pathways; 4 were recruited via National Health Service (NHS) pathways. Adherence was 64%, with higher adherence during phase 1 (81%) than phase 2 (50%). App usage was high (95%), and self-reported program satisfaction was moderate to high. Retention at 24 weeks was 76%, and no adverse events occurred. The qualitative findings supported the program’s feasibility and acceptability, emphasizing social connection and tailored exercises as key to adherence—especially in home-based sessions. Health care professionals identified community organizations and referral pathways as the most practical routes for implementation.

**Conclusions:**

A blended, technology-based DT training program is both feasible and acceptable for older adults at risk of falling and can be effectively delivered beyond clinical settings. Community-based recruitment outperformed NHS pathways, highlighting the value of community engagement. These findings support the feasibility and acceptability of a full-scale trial, with targeted refinements to recruitment, support structures, and delivery to maximize scalability and impact.

## Introduction

Falls and fall-related injuries are a major public health concern. One in three adults over 65 years experiences at least 1 fall each year, and individuals who have already fallen are at an increased risk of falling again [1,2]. Globally, falls are the second leading cause of unintentional injury–related deaths [3], and in the United Kingdom, falls and fall-related injuries cost an estimated £4.4 billion annually [1]. It is estimated that by 2050, 1 in 6 people worldwide will be aged 65 years or older. This demographic shift highlights the urgent need for effective prevention strategies [4,5].

Evidence suggests that falls can be prevented using appropriately prescribed exercise programs. Aging affects not only balance but also cognition, and domains such as attention, memory, and executive function are linked to mobility [6]. Dual-task (DT) training, which combines physical and cognitive exercises simultaneously, has been shown to improve walking speed, executive function, and balance and reduce fall risk in older adults [7,8]. Several lines of evidence suggest that training motor and cognitive function simultaneously, that is, DT training, is superior to single-task (balance or resistance) training or no training for improving mobility, global cognition, and reducing fall rates [9-11], leading to a better quality of life [12]. Training cognition and physical function simultaneously rather than sequentially is more beneficial [13,14]. Simultaneous cognitive and physical training can be delivered via technology (eg, mobile apps), enabling professionals to select cognitive exercises that are suitable to be combined with balance exercises, allowing DT training to be delivered unsupervised at home. Mobile apps are interactive, provide instant feedback, and can send reminders to users. These features promote engagement and adherence to exercise [15-17]. Emerging evidence supports the remote delivery of balance and strength exercise training using technology for falls prevention in older adults [18-20]. For example, a smartphone app delivering an unsupervised, home-based DT program to community-dwelling older adults without a history of falls showed feasibility, high adherence, and improved balance [20]. However, it remains unclear whether older adults with a history of falls, who are at greater risk of recurrence, would be willing or able to engage in an unsupervised, technology-supported DT exercise program. Additionally, the potential role of such programs within existing health care services is uncertain. Current services often cater to frailer individuals with multiple comorbidities who typically require close supervision [21], raising questions about the feasibility of recruitment solely through fall-prevention services as well as whether technology-supported DT training is better delivered in clinical or community settings.

The objectives of this study were to evaluate (1) the feasibility of recruiting and retaining community-dwelling older adults with a history of falls in a blended DT program combining supervised and self-directed sessions supported by technology; (2) the acceptability of the program to participants; (3) its potential effects on mobility, cognition, fall risks (eg, number of falls, fear of falling), quality of life, and use of health care resources; and (4) health care professionals’ perspectives on integrating a blended DT program into existing care pathways for older adults.

## Methods

### Study Design

This was a prospective, single-arm, nonrandomized feasibility study. The study protocol has previously been published [22]. The study is reported according to the CONSORT (Consolidated Standards of Reporting Trials) pilot and feasibility trials checklist (Checklist 1).

### Ethical Considerations

The study received ethical approval from the Health Research Authority East of England – Cambridge East Research Ethics Committee (Reference: 24/EE/0059) and was registered with ISRCTN (ISRCTN15123197; April 16, 2024). All participants provided written informed consent and were assigned a unique study ID to ensure anonymity. Personal data were stored securely on password-protected University of Birmingham servers, with identifiable information accessible only to authorized members of the research team and managed in accordance with the Data Protection Act 2018, UK General Data Protection Regulation, and relevant research governance frameworks. Participants were reimbursed for travel expenses associated with study participation.

### Recruitment

Recruitment took place in 3 periods—May to August 2024, October to November 2024, and March to May 2025—to align with program delivery schedules and public holidays. Participants were recruited through text messages or invitation letters from general practitioners and hospital fall-prevention clinics, and community outreach (eg, retirement villages, charities, interest groups) using open events and study flyers. All participants received the intervention in the gym of the partnering retirement villages and community well-being and leisure centers.

### Participants

The sample size (n=50) was chosen based on the recommendations of published literature for feasibility studies [23-25]. If we approach 100 eligible participants, we will be able to estimate a participation rate of 50% (green stop-go criteria; see Table 1) within a 95% CI of ±9.8%. With a sample size of 50 participants, we will be able to estimate an attrition rate of 20% (green stop-go) within a 95% CI of ±11%. Inclusion criteria included community-dwelling adults aged 65 years or older who had experienced at least 2 falls in the past year (including near misses); had access to a smartphone or tablet; could stand with 1-hand support on their usual walking aid for at least 60 seconds; could stand up from a chair and walk 6 m independently with their current walking aid; were self-toileting; and demonstrated sufficient cognition, hearing, and vision to follow the instructions for the assessment and exercise program. Exclusion criteria included medical conditions precluding safe exercise participation and current participation in another research study involving a fall-prevention intervention. Health care professionals involved in services for older adults within the study regions were recruited to participate in qualitative focus groups or one-to-one interviews.

**Table 1.** Stop-go criteria.

	Criteria
Green	Recruitment ≥50%; adherence ≥80% (ie, completion of at least 58 out of 72 prescribed sessions over 24 weeks); and retention ≥80%. Qualitative data confirm the intervention being feasible and acceptable, with no safety concerns identified.
Amber	Recruitment: 30%-49%; adherence: 50%-79%; and retention: 50%‐79%. Qualitative data indicate that modifications to program design or delivery are needed, but no safety concerns identified.
Red	Recruitment: <30%; adherence: <50%; and retention: <50%. Qualitative data do not support progression to a larger-scale randomized controlled trial and/or indicate unresolved safety concerns.

### Dual Task Exercise Program

All participants underwent the 24-week DT exercise program, consisting of 2 phases:

- Phase 1 (weeks 1‐12): weekly 50-minute physiotherapist-led group sessions (4‐8 participants per group) and two 50-minute home-based sessions, which replicated the class exercises but were completed independently and unsupervised.
- Phase 2 (weeks 13‐24): three weekly 50-minute home-based sessions, guided by a structured exercise plan provided by the research team.

Each session included a 5-minute warm-up, 20-minute DT exercises (static balance or strength exercises while engaging with cognitive games using the commercial *Peak Brain Training* app [26]; Supplementary Material 1 in Multimedia Appendix 1), 20-minute dynamic balance and strength training, and a 5-minute cool-down. The app was used on participants’ smartphones or tablets, positioned on adjustable stands for ease of use.

All participants received a handbook with detailed exercise instructions and had access to online videos demonstrating the balance and strength exercises. Participants recorded weekly adherence in an exercise diary. At the end of phase 1, an in-person education session was provided to reinforce fall awareness and explain the program content in phase 2. Participants could contact the research team for technical support with the app as needed. A TIDieR (Template for Intervention Description and Replication) checklist is presented in Checklist 2. The intervention was co-designed with members of the patient and public involvement and engagement (PPIE) group, particularly regarding the selection of cognitive games to ensure they were appropriate and engaging for older adults. The PPIE group also helped refine program components intended to promote social interaction and engagement, including strategies for maintaining contact during the home-based phase.

### Outcome Measures

#### Overview

The primary outcome measures were the feasibility and acceptability of the program, assessed through:

1. Recruitment rate: calculated as (number recruited via National Health Service [NHS] pathways/number of patients approached by primary and secondary care teams)×100. Recruitment via community pathways was excluded due to unquantifiable outreach.
2. Adherence: calculated as (class attendance +self-reported home-based sessions)/(total prescribed sessions)×100. It was reported separately for phase 1, phase 2, and overall.
3. Retention rate: calculated as (number of participants completing the study/number consented) × 100. Completion was defined as attendance at the postintervention assessment.
4. Acceptability: assessed via a mixed methods exit survey administered at the end of phase 1 (wk 12) and phase 2 (wk 24). The survey comprised structured closed-ended items to capture quantitative data on usability, satisfaction, and perceived effectiveness, alongside open-ended free-text fields designed to elicit qualitative insights into participants’ experiences and perspectives.

#### Stop-Go Criteria

Predefined stop-go criteria [22] were used to provide guidance on whether the project should progress to a full trial with an internal pilot (green), progress to a full trial with an internal pilot with adaptation (amber), or no progression (red; Table 1).

Data were also collected on the following outcomes before and after the intervention. These were possible primary and secondary outcome measures for the main trial, so this enabled the assessment of data collection methods and data completeness of these measures, and exploratory data analysis:

1. Mobility and balance were assessed using the Timed Up and Go (TUG) test and TUG with a DT version of concurrent cognitive task (TUG-Cog), where participants counted backward by 7 from a randomly selected number between 100 and 200. The time taken to complete the test was recorded. All participants first completed 1 familiarization trial, followed by 1 recorded trial for the TUG and 1 recorded trial for the TUG-Cog.
2. Cognitive function was assessed by the total score of the 12-item Everyday Cognition (ECog-12) scales short version [27,28].
3. Fear of falls was assessed by the total score of the Falls Efficacy Scale-International (FES-I) questionnaire [29].
4. Health-related quality of life was assessed using the EQ-5D-5L [30].
5. Usage of health care resources was self-reported using ModRUM [31].
6. Self-reported number of falls in the last 6 months.

TUG and TUG-Cog were assessed in person by an experienced physiotherapist; all other outcomes were collected using a REDCap (Research Electronic Data Capture; v15.4.3) online survey. Survey links were sent to participants 1 week before the intervention and again in week 24, with up to 3 reminders to facilitate response rates. Paper copies were provided to participants upon request.

At baseline, demographic information was collected, including age, sex, ethnicity, level of education, area deprivation (Index of Multiple Deprivation), and type of residence. Frailty was assessed retrospectively using the Clinical Frailty Scale [32] to characterize the participant cohort in this feasibility study and to provide insight into the types of individuals for whom the intervention may be feasible and appropriate. An experienced geriatrician (DW) determined a frailty score following a detailed review of the demographic and functional data alongside a discussion with the team using the Clinical Frailty Scale [33].

### Qualitative Methods

#### Focus Groups

Ten focus group semistructured interviews with 28 participants who completed the DT program (2‐5 participants per group) were conducted. The focus groups were constructed to provide explanatory data on the study objectives (Supplementary Material 2 in Multimedia Appendix 1). Additionally, 4 online focus groups (n=12) and 4 online one-to-one interviews (n=4) were conducted with health care professionals (12 were physiotherapists, 1 occupational therapist, 1 general practitioner, 1 consultant geriatrician, and 1 research nurse) using a semistructured interview guide (Supplementary Material 2 in Multimedia Appendix 1). Focus groups were led by a member of the research team (PM), took place at the same venues as the exercise classes, were audio-recorded, and later transcribed verbatim. Online interviews were conducted using Microsoft Teams and were transcribed using the Microsoft Teams function. The interviews with the participants and health care professionals lasted between 45 and 80 minutes and 25 and 70 minutes, respectively.

#### Exit Surveys

Data were also collected from participants at the end of each program phase using an exit survey, which included both quantitative satisfaction ratings (Likert-scale items) and qualitative feedback from 8 open-ended questions. The questions were related to perceived benefits from the program, the experience of using the PEAK app while dual tasking, and recommendations for improving program delivery (Supplementary Material 3 in Multimedia Appendix 1).

### Qualitative Data Analysis

Data from the focus groups and exit surveys were organized using NVivo 14 (Lumivero). Framework analysis was applied to the dataset to guide the examination of the study objectives, while also allowing for the identification of unanticipated themes and insights that emerged during the analytical process [34]. The data were initially coded deductively by members of the research team (PM, AS, and SYC) using a set of predefined categories that had been agreed upon in advance. These categories were directly aligned with the study objectives and included engagement and adherence, app usability, and retention. Once the data were organized according to these categories, an inductive approach was used to explore and identify additional factors contributing to each of the main categories. The coding process was led by PM and SYC, and a deliberative strategy [35] was used to enhance rigor. This involved multiple researchers (PM, SYC, AS, VAG) collaboratively reviewing and discussing the codes to refine interpretations and develop overarching themes. PPIE members also contributed to the interpretation of the focus group findings, providing perspectives that helped contextualize participants’ experiences and identify practical implications for program delivery.

The explanatory themes were organized within the main categories identified through the framework analysis, which aligned with the study’s objectives. These qualitative findings were then integrated with the descriptive quantitative analysis during the reporting phase. To ensure analytical rigor, a relativist approach to quality was applied, guided by key criteria including breadth, credibility, and coherence in the study design [36].

### Quantitative Data Analysis

The analysis undertaken within this feasibility study was mainly descriptive. The progression criteria in the stop-go were summarized as proportions or means along with 95% CIs. The number of days of exercise and the number of physical exercises and cognitive games completed and recorded in the exercise diaries during the program were summarized to describe the adherence and usage of the app. To understand any change in the usage of the app between phase 1 and phase 2, we assessed the number of cognitive games completed in phase 1 and phase 2 separately.

The data collected on proposed outcome measures (eg, TUG) were summarized at baseline and follow-up using appropriate summary statistics. Exploratory analysis using a paired *t* test or Wilcoxon signed-rank test (depending on the distribution of the data) was undertaken to provide an exploratory assessment (using CIs; no *P* values are reported) of the effect of the blended intervention. All analyses were conducted in IBM SPSS Statistics (version 29; IBM Corp., 2022).

## Results

### Participant Recruitment

**Figure 1.**
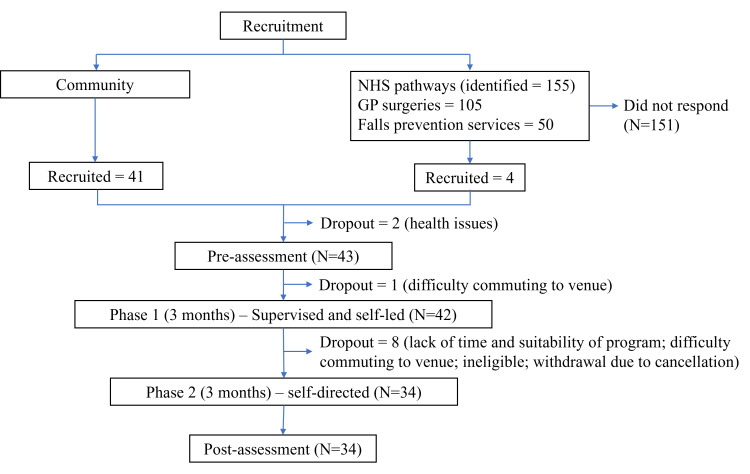
Flowchart. GP: general practitioner; NHS: National Health Service.

Forty-five older adults were recruited: 41 (91%) from community sources and 4 (9%) through NHS pathways (Figure 1). Community recruitment involved 10 retirement villages, 19 local activity groups, 8 instructors, and the Birmingham 1000 Elders mailing list, using open events and flyer distribution. As these approaches were open-access and did not involve direct invitations or approaches, it was not possible to determine the number of individuals exposed to the study information or formally approached. Therefore, a conventional recruitment rate (approached vs consented) could not be calculated for the community pathway. In the NHS recruitment pathways, 155 potential participants were identified through general practitioner surgeries (n=105) and secondary care falls clinics (n=50), but only 4 were enrolled (2.6%; 95% CI 0.7%‐6.5%), meeting the red stop-go criteria (Table 2). Nonparticipation was mainly due to health concerns, travel distance, or lack of a suitable device (eg, smartphone).

**Table 2.** Feasibility study outcomes and the stop-go criteria.

Outcome	Values; stop-go criteria	Full stop-go criteria
Recruitment rate from the NHS^a^ pathways	Proportion 3% (95% CI 1%‐7%); red (red to red)	Green: ≥50%; amber: 30%‐49%; red: <30%
Adherence	Median 64% (IQR 46%‐82%; 95% CI 53%‐72%); amber (amber to amber)	Green: ≥80%; amber: 50%‐79%; red: <50%
Retention rate	Proportion 76% (95% CI 60%‐87%); amber (amber to green)	Green: ≥80%; amber: 50%‐79%; red: <50%

a NHS: National Health Service.

**Table 3.** Median adherence in each phase and overall.

Phase	Number of actual exercise days, median (IQR; 95% CI)	Adherence (%), median (IQR; 95% CI)
Phase 1	27 (24‐31; 22‐29)	81 (67‐87; 64‐82)
Phase 2	18 (14‐21; 14‐23)	50 (17‐83; 39‐65)
Phase 1+2	46 (33‐57; 37‐51)	64 (46‐82; 53‐72)

**Table 4.** Number of physical exercises and cognitive games completed (in each phase and overall).

Phase	Number of physical exercises completed, median (IQR; 95% CI)	Number of cognitive games completed, median (IQR; 95% CI)
Phase 1	156 (122‐148; 123‐167)	78 (69‐92; 63‐85)
Phase 2	137 (73‐190; 94‐162)	67 (36‐96; 44‐80)
Phase 1+2	275 (194‐338; 224‐322)	133 (98‐174; 111‐161)

The program consists of 3 exercise days per week in each phase (12 weeks); the total number of prescribed exercise days is 36 in each phase (Table 3). Participants are prescribed 6 physical exercises and 3 cognitive games in a week in each phase; the total number of prescribed physical exercises and cognitive games in each phase is 216 and 108, respectively (Table 4).

### Sample Characteristics

The mean age of the participants was 78.3 (SD 7.6) years, and the majority were female participants (n=31, 68.9%). The number of participants recruited from sheltered and private accommodation was 13 (28.9%) and 32 (71.1%), respectively. The median Index of Multiple Deprivation score was 4 (IQR 1-10). The majority of the participants were White British (n=42, 93.3%). All participant characteristics as well as the characteristics of participants contributing to the qualitative study are presented in Table 5.

**Table 5.** Participant characteristics for all participants (N=45) at baseline and for participants (n=28) who took part in the focus groups.

Characteristics	All participants	Participants in focus groups
Age (y), mean (SD)	78.3 (7.6)	78.1 (8.9)
Sex, n (%)		
Female	31 (68.9)	20 (71.4)
Sheltered residents, n (%)	13 (28.9)	10 (35.7)
Private residents, n (%)	32 (71.1)	18 (64.3)
IMD^a^ scores, median (IQR)	4 (1-10)	4.5 (1-9)
Highest level of education, n (%)		
Secondary education	9 (20)	7 (25)
Further education	6 (13.3)	4 (14.3)
Higher education	29 (64.4)	17 (60.7)
Prefer not to say	1 (2.2)	0 (0)
Ethnicity, n (%)		
White British	42 (93.3)	25 (89.3)
Asian or Asian British	3 (6.7)	3 (10.7)
Participant-reported data		
TUG^b^ (s), mean (SD)	11.1 (3.5)	11.1 (4.0)
TUG-Cog^c^ (s), mean (SD)	15.1 (7.2)	14.5 (6.0)
ECog-12^d^, mean (SD)	1.61 (0.4)	1.6 (0.4)
Clinical frailty score, median (IQR)	3 (2-6)	3 (2-5)
FES-I^e^, mean (SD)	31.8 (10.4)	32.9 (9.7)
High concern, n (%)	25 (59.5)	18 (64.3)
Moderate concern, n (%)	14 (33.3)	10 (35.7)
Low concern, n (%)	3 (7.1)	0 (0)
Falls in last 6 months, median (IQR)	2 (0‐8)	2 (0‐8)
EQ-5D-5L VAS score (out of 100), mean (SD)	72.1 (18.0)	74.1 (16.8)
EQ-5D-5L score, mean (SD)	0.644 (0.207)	0.655 (0.151)
Number of A&E^f^ visits (sum)^g^	19	14

a IMD: Index of Multiple Deprivation.

b TUG: Timed Up and Go.

c TUG-Cog: Timed Up and Go test with a concurrent cognitive task.

d ECog-12: The Everyday Cognition scales short version.

e FES-I: Falls Efficacy Scale International.

f A&E: accident and emergency.

g This variable corresponds to the first item of the Modular Resource-Use Measure; the full results are reported in Supplementary Material 5-6 in Multimedia Appendix 1.

### Study Retention

Thirty-four of the 45 participants completed the study, giving a retention rate of 76% (95% CI 60%‐87%), which met the amber stop-go criteria (Tables 2-4). A total of 11 participants did not complete the trial. Three withdrew after providing consent but before commencing the intervention due to health issues (n=2) and difficulty commuting to the venue where exercise classes were scheduled to take place (n=1). A further 8 participants withdrew during the intervention phase. Five withdrew due to time constraints or because they felt the program was not suitable for them. One participant was later deemed ineligible due to severe balance issues. Two additional participants were recruited during a third recruitment round; however, one of them withdrew after the first session because the venue was too far from their home. As this left only 1 participant in that group, the planned group sessions could not proceed, and the final participant was therefore withdrawn from the study (Figure 1).

### Adherence and Acceptability of the Intervention

Overall adherence to the program was 64% (95% CI 53%‐72%), meeting the amber stop-go criteria (Tables 2-4). On average, participants completed 46 days (95% CI 37‐51) of combined cognitive and physical exercises out of the 72 days prescribed. Of the 432 prescribed physical exercises and 216 prescribed cognitive games, participants completed 275 physical exercises (64%, 95% CI 224‐322) and 133 cognitive games (62%, 95% CI 111‐161), respectively. These results indicate that participants engaged with and completed the intervention largely as intended. Adherence was higher in phase 1 (27 out of 26 days, 81%), which combined supervised and self-directed sessions, compared with phase 2 (18 out of 36 days, 50%), which was entirely self-directed. Within phase 1, supervised class attendance was 81% (9 out of 12 classes), and adherence to home-based sessions was 79% (18/24 home sessions). Difficulty finding time for weekly exercises was noted by 17% (n=5) of the participants in phase 1 and 41% (n=12) in phase 2. Adherence to the PEAK app was 95% (n=32). Participants valued its flexibility (n=23, 69%), although the proportion reporting ease of use declined from 59% (n=17) at the end of phase 1 to 34% (n=10) at the end of phase 2. No adverse events were reported.

The results of the exit survey highlighted that the median perceived program benefit was 7 out of 10 (1=not beneficial; 10=very beneficial). To explore whether perceived benefit influenced adherence, participants were divided into 2 groups based on this median score. Those who rated the program as highly beneficial (score ≥7) demonstrated higher adherence (n=15, 83%; median 33.3, IQR 63.9-97.2) compared to those with lower perceived benefit (score <7; n=13, 36%; median 27.8, IQR 16.7-44.4). Just over half (52%) of the participants agreed that integrating the app into the blended digital therapy program was a good way to improve balance. The majority (n=25, 86%) expressed satisfaction with the support received from both the research team and the physiotherapist. On average, participants reported a 6 out of 10 likelihood of continuing the program after the study concluded. The full results from the exit survey are available in Supplementary Material 3 in Multimedia Appendix 1.

**Figure 2.**
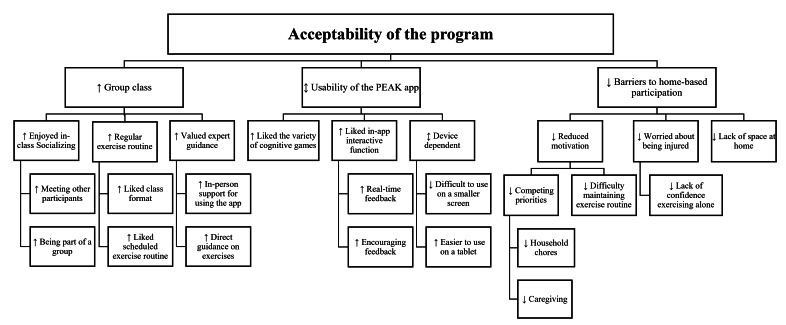
Thematic tree from the focus group with participants. Symbols indicate positive (↑), negative (↓), and neutral (↕) feedback.

Qualitative findings reinforced the quantitative results, highlighting social interaction as key to participant engagement (Figure 2—thematic tree). Group classes were valued for structure, motivation, enjoyment, and social connection, while the unsupervised phase saw reduced motivation and adherence due to competing priorities. Participants appreciated app features such as age-based rankings and the variety of cognitive games, though some struggled with small smartphone screens. Barriers to home exercise included limited space, low confidence, inappropriate difficulty levels, and concerns about joint pain. Illustrative quotes are provided in Supplementary Material 4 in Multimedia Appendix 1.

Participants appreciated the feedback features (eg, ranking within age group, positive reinforcement) of the app and the variety of cognitive games, many of which were perceived as appropriately challenging. Challenges included difficulty using the app on smaller screens of smartphones.

### Exploratory Analysis of Proposed Outcome Measures

The TUG is the planned primary outcome for the proposed future trial. Both TUG and TUG-Cog scores decreased after the program (Table 6). The FES-I scores decreased following the program, suggesting a reduction in fear of falling; changes in the category of the FES-I [29] at postassessment are presented in Table 7. There was no change in ECog-12 scores, but there were decreases in EQ-5D-5L tariff and VAS scores (Table 6). The completion rates for TUG and TUG-Cog were 93.94%, and for ECog-12, FES-I, Modular Resource-Use Measure, and EQ-5D-5L, the rates were 98.5%.

**Table 6.** Exploratory data analysis of proposed outcomes for the full trial.

Outcomes	Preassessment	Postassessment	95% CI of the difference
TUG^a^ (s; n=31), mean (SD)	10.32 (4.17)	8.80 (5.74)	−0.94 to 0.99
TUG-Cog^b^ (s; n=31), mean (SD)	12.13 (7.66)	11.51 (8.52)	−1.90 to 0.94
ECog-12^c^ (points, n=33), mean (SD)	1.57 (0.36)	1.52 (0.39)	−0.21 to 0.48
FES-I^d^ (points, n=33), mean (SD)	29.50 (11.25)	25.00 (9.75)	−5.50 to −1.50
High concern, n (%)	20 (60.61)	14 (42.42)	—^e^
Moderate concern, n (%)	12 (36.36)	13 (39.39)	—
Low concern, n (%)	1 (3.03)	6 (18.18)	—
Number of falls in last 6 months (n=33), median (IQR)	2 (0‐8)	0 (0‐10)	—
EQ-5D-5L^f^ tariff (n=33), mean (SD)	0.675 (0.191)	0.652 (0.212)	−0.316 to 0.367
EQ-5D-5L VAS (n=33), mean (SD)	73.79 (16.91)	71.30 (17.54)	−0.13 to 0.56
Number of A&E^g^ visits (sum, n=33)^h^	16	10	−0.5 to 0.00

a TUG: Timed Up and Go test (seconds; lower scores indicate better mobility).

b TUG-Cog: Timed Up and Go test with a concurrent cognitive task (seconds; lower scores indicate better dual-task performance).

c ECog-12: The Everyday Cognition scales short version (higher scores indicate greater perceived cognitive difficulty).

d FES-I: Falls Efficacy Scale International (higher scores indicate greater fear of falling).

e Not applicable.

f Higher scores indicate better health-related quality of life.

g A&E: accident and emergency.

h This variable corresponds to the first item of the Modular Resource-Use Measure; the full results are reported in Supplementary Material 5-6 in Multimedia Appendix 1.

**Table 7.** Changes in the category of Fall Efficacy Scale–International (FES-I)^a^.

FES-I cutoff scores	Postassessment		
	High	Moderate	Low
Preassessment			
High^a^	11	2	7
Moderate	0	1	0
Low	3	3	6

a High concern: 28‐64; moderate concern: 20‐27; low concern: 16‐19 [29].

### Implementation of the Intervention in Health Care and Community Pathways

**Figure 3.**
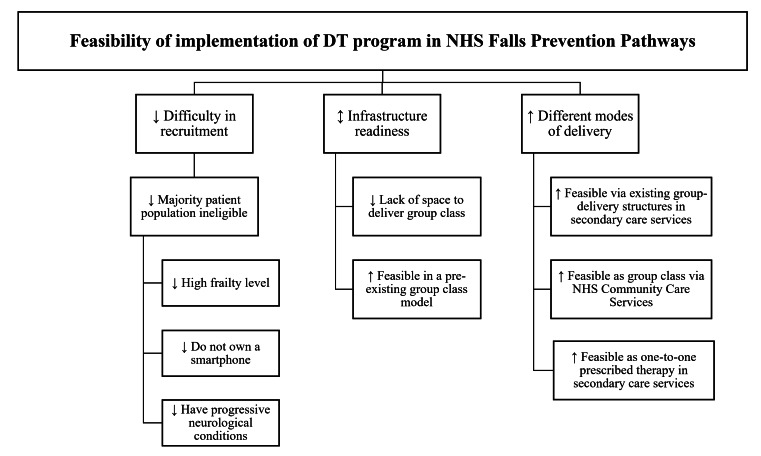
Thematic tree from the focus group with health care professionals. Symbols indicate positive (↑), negative (↓), and neutral (↕) feedback. DT: dual-task; NHS: National Health Service.

A thematic tree illustrating the qualitative results is presented in Figure 3. Health care professionals viewed recruitment from the NHS as challenging, as many patients attending fall-prevention clinics were too frail, had comorbidities (eg, syncope), lacked the capacity for unsupervised balance exercises, or did not have access to suitable devices for cognitive training. Several suggested widening recruitment beyond falls services to include people under 65 years or those beginning to notice balance difficulties, allowing issues to be managed earlier.

Implementation was seen as dependent on hospitals. Hospitals already offering group classes were viewed as best suited for integration, while those without such provisions were less likely to consider adopting the program. One-to-one delivery was seen as a possible option if supported by evidence and a wider range of exercise levels to enable personalization. Many professionals also emphasized that delivery through community organizations, accessed via health referral pathways, would reduce travel demands for older adults. Qualitative quotes are in Supplementary Material 4 in Multimedia Appendix 1.

## Discussion

### Principal Findings

This study demonstrates that a blended, technology-supported DT training program can be both feasible and acceptable for older adults who have experienced at least 2 falls in the past 12 months (including near misses). The combination of supervised group sessions and self-directed home training was well received, with digital engagement enhanced by appropriate support. With design modifications to improve adherence and retention, DT training can be effectively delivered in community settings, representing a shift from clinic-based rehabilitation models that typically rely on one-to-one supervised, therapist-led exercise interventions [37].

The study’s key contribution lies in demonstrating that complex interventions, such as DT training, can be adapted for scalable, community-based formats that integrate digital health technologies. This approach has the potential to extend the reach of evidence-based fall-prevention strategies and support their integration into routine community health care pathways.

### Participant Recruitment

Recruitment via NHS fall-prevention and primary care pathways—using emails, letters, or texts—was difficult and met the red stop-go criteria. This aligns with prior trials on the physical health of older adults reporting low general practitioner recruitment rates (~6%) [38-40]. Recruiting older adults into exercise studies is widely recognized as challenging. Many older adults experience complex health needs, chronic pain, and multiple comorbidities, all of which may limit their capacity or willingness to participate in research [41]. In our study, health care professionals within NHS Falls Prevention Services also reported a limited pool of eligible participants. Many of their patients were frail, dependent on caregivers, or living with progressive neurological conditions, such as dementia, which restricted their ability to safely participate in group-based or self-directed, unsupervised exercise programs. The literature further highlights several barriers to recruiting older adults into exercise studies. These include social stigma associated with programs targeted at “older people” or “ fallers”, logistical barriers such as transportation, concerns about the demands or expectations of participation [42-44], and protective attitudes from family members [41].

To address these challenges, we suggest several strategies for future trials. These include working with patient groups to develop more acceptable and inclusive language when describing the study, forming partnerships with community organizations to help address logistical barriers, and framing study aims in ways that appeal to older adults with diverse motivations for participation.

Limited contact (a single invitation) and ethical restrictions on reminders likely contributed to our low response rate. Evidence supports the use of multiple prompts [41], and Cochrane reviews recommend telephone follow-up reminders in low-response trials [42].

Despite limited success with NHS-based recruitment, the study did enroll 45 of the planned 50 participants (90% of the target), the majority (41/45) through community-based strategies. Face-to-face events and flyers distributed via local organizations were more effective, with in-person engagement supporting informed decision-making—especially important for digital interventions unfamiliar to some older adults [45].

Although NHS-based recruitment met the red stop-go criteria, the study still achieved 90% of its recruitment target, primarily through community routes—demonstrating feasibility for a larger trial. Expanding recruitment to include services such as frailty front door services and fracture liaison services could enhance reach and inclusivity.

### Study Retention

Retention met the amber stop-go criteria, indicating a need for trial design modifications. The main reasons for withdrawal were the distance of class venues from participants’ homes and perceptions that the program was not sufficiently tailored to individual needs. Classes were scheduled at different times of the day but were restricted to a single day each week due to limitations in staff availability and venue costs. Increasing delivery resources and expanding venue options could improve accessibility and retention [46]. Additionally, incorporating greater flexibility and personalization may boost relevance and continued engagement [47].

### Adherence and Acceptability of the Intervention

Adherence met the amber stop-go criteria, indicating the need for trial design modifications—particularly for the unsupervised home-based phase. Consistent with previous evidence, adherence to home-based exercise was lower compared with the group-based programs, particularly when structured supervision and support were reduced [38,48-50]. For comparison, a meta-analysis of community-based group exercise reported an average adherence of 69% (SD 14.6) [51]. Participants valued the motivation, enjoyment, and accountability of supervised group classes but reported reduced motivation, competing priorities, and difficulties sustaining routines once the program became self-directed. Other programs with ongoing support (eg, coaching [52], follow-up calls [19]) have achieved higher adherence rates (75% and 85%, respectively).

Social interaction emerged as a key driver of engagement with participants, suggesting that peer-support groups; virtual group sessions; and reminders via text, phone calls, or app notifications could help support longer-term engagement. Additionally, in-app feedback and a sense of progress also supported adherence [53].

These findings highlight that continuous support, rather than delivery format alone, is key to sustaining engagement among older adults. While supervised sessions help build initial confidence, long-term adherence depends on ongoing social interaction, feedback, and motivational support. Incorporating continuous support mechanisms—delivered in person, virtually, or through structured follow-ups—can help maintain participation and maximize program effectiveness over time.

### Implementation of the Intervention in Health Care and Community Pathways

Health care professionals’ views on implementing the DT program within NHS services were shaped by patient profiles and infrastructure. According to National Institute for Health and Care Excellence guidelines on falls assessment and prevention, patients referred to fall-prevention clinics are typically frail, present with multiple comorbidities, or have impaired mobility [21]. These individuals often require close supervision (eg, one-to-one delivery) and are therefore not well suited to a program that includes group-based and unsupervised components. The findings from the qualitative data suggest that clinicians in fall-prevention services tended to view this supervised and self-directed technology-based DT program as more appropriate for preventive care in at-risk populations, rather than as a treatment option for those already attending specialist services.

While established programs (eg, Otago and Falls Management Exercise) primarily target physical components of falls risk (eg, strength and balance), the DT program additionally trains executive functions such as attention allocation and task switching during movement—skills that are important for navigating real-world environments where falls often occur during multitasking. This approach may complement existing programs by addressing cognitive-motor demands that are not explicitly targeted in the existing interventions.

In contrast, clinicians working in preventive pathways reported that many of their patients could benefit from the program and were more receptive to its integration into the NHS. Infrastructure was also identified as a key factor influencing implementation. The findings from the qualitative interviews indicated that a lack of space for group classes was commonly cited by health care professionals as a barrier to implementation, whereas services with existing exercise programs expressed greater readiness to adopt the intervention (Figure 3). While the intervention in this feasibility study was delivered by physiotherapists from NHS Falls Prevention Services, health care professionals indicated that, in routine practice, the program could be delivered by appropriately trained exercise professionals, such as therapy assistants or support workers, personal trainers, and qualified community-based exercise instructors. Embedding the program within primary care and community-based infrastructures—such as Primary Care Network referral pathways, social prescribers, local support groups, and leisure providers—offers a scalable, sustainable model aligned with evidence supporting high-dose, balance-challenging exercise [54] and may enhance accessibility and adherence through community engagement and health care referral pathways. These settings often already use qualified instructors and provide accessible venues, which may facilitate wider reach and long-term delivery.

### Strengths and Limitations

A key strength of this feasibility study was its mixed methods approach, which added valuable context to the quantitative outcomes. High completion rates of the exploratory outcome measures among participants who completed the study (>90%) support the suitability of the data collection methods used in this study for older adults. However, the participants recruited lacked ethnic diversity and were skewed toward participants with higher education. While exercise frequency was captured via participant diaries, we did not collect data on exercise intensity (eg, heart rate, perceived exertion) or exercise quality during independent home sessions. This limits our ability to fully assess intervention fidelity and determine whether differences between phases may have been influenced by variations in exercise dose or execution. Future trials should incorporate objective or structured monitoring of home-based exercise intensity and quality to strengthen fidelity assessment. Furthermore, conducting the study exclusively in English—due to funding constraints—also limited inclusivity. Future trials should consider translation services to improve representativeness and reach.

### Conclusions

This feasibility study shows that a blended DT training program is acceptable to older adults and can be delivered outside clinical settings. Adherence declined when participants were without direct supervision, highlighting the importance of ongoing support and social connectedness for sustained engagement. Community-based recruitment outperformed NHS pathways, and health care professionals identified community organizations and referral schemes as the most viable implementation channels. Overall, these results support progression to a full trial with adaptations to recruitment strategies, support mechanisms, and delivery pathways to enhance feasibility and scalability.

## Supplementary material

10.2196/87577 Multimedia Appendix 1 List of cognitive games, focus group/interview schedules, exit surveys results, qualitative data with quotes, ModRUM results, and unit costs used in the calculation of ModRUM results.

10.2196/87577 Checklist 1 CONSORT 2010 checklist.

10.2196/87577 Checklist 2 TIDieR checklist.

## Abbreviations

CONSORT: Consolidated Standards of Reporting Trials
DT: dual-task
FES-I: Falls Efficacy Scale-International
NHS: National Health Service
PPIE: patient and public involvement and engagement
REDCap: Research Electronic Data Capture
TIDieR: Template for Intervention Description and Replication
TUG: Timed Up and Go
