# Supplementary material for: Supervised and Self-Directed Technology-Based Dual-Task Exercise Training Program for Older Adults With a History of Falls: Mixed Methods Feasibility Study
Source: JMIR Aging. 2026 May 18;9:e87577. doi: 10.2196/87577 (PMC13183345; doi:10.2196/87577)
Supplement: Multimedia Appendix 1 [file aging-v9-e87577-s001.docx]

**Supplementary material 1.** List of prescribed cognitive games from the PEAK app.

1. Games under the *Focus* domain
   1. Must Sort
   2. Rush Back
   3. Unique
   4. Decoder
2. Games under the *Memory* domain
   1. Perilous Path
   2. Memory Sweep
   3. Spin Cycle
   4. Apprentice Wizard
   5. Partial Match
3. Games under the *Mental Agility* domain
   1. True Color
   2. Face Switch
   3. Refocus
   4. Turtle Traffic
4. Games under the *Problem Solving* domain
   1. Puzzle Box
   2. Slider
   3. Low Pop
   4. Castle Block
   5. Earth Defence

**Supplementary material 2. Interview schedules**

**Technology-based dual-task training in older adults**

Focus group schedule for participants.

Version 1 28/02/2024

1. **Introduction by Moderator (5 minutes)**

Thank you all for joining us today. We really appreciate your time and willingness to share your experiences from the "Feasibility and Acceptability of a Supervised and Self-Directed Technology-Based Dual-Task Training Programme for Older Adults at Risk of Falling” study. Your feedback is invaluable in helping us understand what worked well and how we can improve the programme. The purpose of this session is to hear your thoughts on participating in the programme over the past six months. There are no right or wrong answers—we’re here to learn from your experiences. The discussion will take about 60 minutes and will be recorded to ensure we capture everything accurately.

**To protect your privacy:**

- Your names will not appear in any reports.
- If we use quotes, we’ll use pseudonyms.
- The recordings will only be accessible to the research team, stored securely on the University research data server for 10 years, and then deleted.

**A few ground rules before we begin:**

- Please avoid using real names during our discussion; if you do, we’ll replace them with pseudonyms in the transcript.
- Let’s aim for a respectful and collaborative conversation. It’s okay to agree or disagree, but please allow one person to speak at a time so everyone can share their thoughts.

Now, let’s start by introducing ourselves. Please share your first name or a nickname you’re comfortable using today.

Before we continue, I’d like to confirm that you all agree to keep this discussion confidential and that you’re happy to proceed. If so, please raise your hand to indicate your consent.

Thank you! I’ll now begin the recording.

1. **Study design, conduct and processes (15 minutes).**

Thank you for introducing yourselves. Let’s start by talking about the overall design and processes of the study. I’ll ask a few questions to understand your experiences.

1. **Expectations before the study**

- What did you expect when you first agreed to participate in this study? (*prompt: Did you hope to get out more, meet new people, improve your mobility, attend exercise classes, or anything else?*)
- Do you feel that the study met these expectations?

– If yes, in what way did it meet your expectations?
– If no, what was different to what you had expected?

1. **Enjoyable and Challenging Aspects**

- What did you enjoy the most about participating in the study?
- What did you enjoy the least, or find challenging?

1. **Recruitment process**

- How did you first hear about the study? *(Prompt: Was it through a flyer in your community, a recommendation from someone, or another way?)*
  - *For people who self-referred:* What made you decide to contact us and take part in the study? *(Prompt: Was it the information on the flyer, curiosity about the programme, or something else?)*
  - *For people who were referred by their GPs or physiotherapists:* how did this influence your decision to participate?
- How did you find the process of getting in touch and signing up for the study? *(Prompt: Was it straightforward, or were there any challenges? How did you find the Open Day at the village?)*
- Looking back, do you think there’s anything we could do differently to reach to more people and to make it easier for people to join the study in the future?

1. **Programme content and delivery (15 minutes)**

Let’s talk about the different aspects of the programme, including the content and how it was delivered.

1. **Group Classes once a week, plus two exercises at home:**

- How did you find your improvement/progression from the classes? *(Prompt: did you feel there was enough variety of balance exercises for you to progress and improve on?)*
- How did you find the way it was delivered, as in length/structure of the class, support given, physio-led? (*Prompt: how important it is for you to a class like this to be led by a physiotherapist or could be a qualified instructor?*)

1. **Home-based Exercise**

- Home exercise twice in a week alongside the group classes: how did you find the handbook? Did it have sufficient information for you to follow and exercise at home? Was the frequency manageable for you?
- Pure home exercise three times in a week: how did you find going through the exercise by yourself for 12 weeks? Was the frequency manageable for you?

1. **Technology Use**

You have provided your feedback to the PEAK app in the EXIT questionnaire. We have some specific questions related to the feedback.

- How did you find the variety of the games? (*Prompt: did you feel that the games were suitable to be used with balance exercise? Did you feel that you were introduced too many games, or not enough?*)
- Did your perception of the PEAK app change between the first part (group classes) and the second part (home-based exercises) of the programme?
- After completing such a programme involving use of app/technology, what role did it play in your relationship with other technologies? (*Prompt: since then did you try new apps or technologies?*)

1. **Home-based vs. In-Person Components**

- What role did the in-person group classes play in your experience with the programme? Did they affect your ability or confidence to continue exercising during the home-based phase? If so, how?
- How did you find the balance between home-based exercise (the 2^nd^ part) vs. in-person group classes (the 1^st^ part)?
- (*We know that home-based exercises are difficult and less motivated from the EXIT questionnaire*) How could an in-person class to support you engaging the home-based exercises?

1. **Peer support**

- In phase 2, how did you find having peer support to help keep you motivated to stay in the programme? *(Prompt: Did you meet other people in the programme? Did connecting with others make a difference?)*

1. **Usefulness**

- What did you like/dislike about the programme? What aspects of the programme were most useful to you?

1. **Measures (e.g., are the process valid for the service users?) (10 minutes)**

Now, let’s discuss the assessment items used in the study (remind participants that they were assessed three times: before, at mid-point of, and after training).

- What do you think about the tests/questionnaires you were asked to do/fill in?
  (*Prompt: Did you feel they were relevant to you?*)
- Were these assessments easy to understand?
- Did you find them burdensome or manageable to complete?
- Is there anything we could do to make the assessments easier or more relevant for you and people like you in the future?

1. **Outcomes (10 minutes)**

Finally, let’s talk about the impact of the programme and any suggestions you may have.

- Have you noticed any changes in you as a result of participating in the programme?
- What do you think about this programme as part of the NHS care? When do you think it should be introduced to people like you and who should be introducing the programme to patients? (GPs or physiotherapists, etc)

1. **Closing (5 minutes)**

Before we wrap up, I’d like to ask:

- Would you participate in this programme again/ or recommend this to a friend, and can you explain why/why not?
- Is there anything we haven’t discussed that you would like to add and that is important for us to know about the experiences of participating in this programme?

Thank everyone for their time and useful participation.

**Technology-based dual-task training in older adults**

Stakeholder focus group schedule

Version 2 01/07/2024

1. **Introduction by Moderator**

Thank you very much for agreeing to speak to me about the “Feasibility and Acceptability of a Supervised and Self-Directed Technology-Based Dual-Task Training Programme for Older Adults at Risk of Falling” study, we really appreciate your time. The reason for this meeting is to discuss the study, so that we can see what is working well and how we can improve the Technology-Based Dual-Task Training programme. There are no right or wrong answers. The discussion will take approximately 40 minutes and will be audio recorded to ensure that we do not miss anything.

We will not use your name in any of our reports. If we do use any quotes from you, we will use a false name. The recordings will only be available to the small team of researchers on this project. The recordings and transcriptions will not be stored with your name on, so no-one will be able to connect you to what you say. We will store the recordings and the transcriptions on a secure server for 10 years after which they will be destroyed.

Let’s aim for a respectful and collaborative conversation. It’s okay to agree or disagree, but please allow one person to speak at a time so everyone can share their thoughts.

1. **Summary of results and participant feedback**

A summary of the study using a PowerPoint Presentation: We wanted to study the feasibility and acceptability of Technology based Dual Task programme for older adult at risk of falling. Research shows that the inability to multitask is associated with a higher fall risk as both cognitive and physical functions play a role in maintaining balance. So, we developed a blended supervised and self-directed technology-based training programme, where participants first undergo 12 weeks of physiotherapist-led group exercises combining balance and cognitive tasks using a mobile app, followed by 12 weeks of self-directed home-based training.

1. **Recruitment, eligibility criteria**
   1. Who would you prescribe this programme to? Whom do you think it is suitable for? (*prompt: ability of the patient to do the training programme*)
   2. From the patients that you see in your clinics, are there any suitable for this programme?
      1. If yes, how many patients, that you see on a daily basis, would be suitable for the Training Programme? (*If they say very little, then why so? If yes, why was it so difficult for us to recruit from the clinics?*)
   3. Where do you think the programme should sit so that it can reach more patients who can benefit from it?
      1. What do you think about the programme being embedded in your own practice?
      2. Which pathway would be better to recruit from?
      3. Which team would be best to deliver the programme?
2. **Programme and content delivery**
   1. What do you think about the blended delivery (combination of supervised and unsupervised sessions) of the programme? What are the strengths and limitations of our training programme? *(Prompts:*
      1. *How could the quality of the programme be enhanced?*
      2. *participants suggested that they would either like for us to put them in peer support groups or just call/text them every now and then to remind them to do the exercises. They would also like longer classes (1hr 30 min) and have more repetitions of the exercises and then have time to mingle amongst themselves. They say that this might help them to stay motivated to increase their adherence. Would NHS have the resources to implement this or community services can address these concerns better?*
      3. *If it is difficult to control for the quality of exercises in a class, then can you provide one-on-one sessions to your patients? (could you include dual task exercises in your one-to-one session with the patient or is there any other way to design it).*
   2. Who would be the best person to deliver the exercise classes? (*Prompt: for this study, we had a Band 6 Physio deliver the classes. Some participants said that they would be happy for a qualified instructor who can answer their questions or address concerns related to the exercises, who isn’t necessarily a Physio. What do you think?*)
3. **Closing**
   1. Is there anything that we haven’t discussed that you would like to ask and that is important for us to know about this programme?
   2. Thank everyone for their time and useful participation.

**Supplementary material 3. EXIT surveys**

| Items (number of data = 29) | Week 12 | | | Week 24 | | |
| --- | --- | --- | --- | --- | --- | --- |
| 1. Overall, how beneficial did you find the training to be? (on a scale of 1 to 10) | 8 (4-10) | | | 7 (5-10) | | |
| 1. How easy was it to fit the training into your daily routine? (on a scale of 1 to 10) | 7 (2-10) | | | 6(1-10) | | |
| 1. Which of the following did you enjoy about the app? (could choose more than one) |  | | |  | | |
| Fun to do | 14 | | | 14 | | |
| Easy to setup | 17 | | | 10 | | |
| Ability to do it at home, any time I want | 23 | | | 20 | | |
| Sense of achievement after doing it | 13 | | | 12 | | |
| Energize me and uplifting my spirit | 9 | | | 11 | | |
| Other - please specify | 14 (Generally enjoyable, motivating, challenging) | | | 10 (Enjoyable, fun, rewarding) | | |
| 1. Which of the following limits your use of the app? (could choose more than one) |  | | |  | | |
| Difficult to set up the device for exercise | 2 | | | 1 | | |
| Exercise is too easy for me | 1 | | | 2 | | |
| Can't find a space to do it | 2 | | | 3 | | |
| Didn't enjoy the activities/ instructions | 0 | | | 3 | | |
| Lack of variety in exercises | 4 | | | 3 | | |
| Just forgot | 5 | | | 4 | | |
| Didn't feel enough progression /benefits through the exercise | 6 | | | 3 | | |
| Didn't have time to do all the exercises in a week | 5 | | | 12 | | |
| Exercises too physically challenging | 5 | | | 1 | | |
| Other - please specify | 9 (technical difficulties such as no internet) | | | 10 (difficulty navigating app, health issues, competing priorities) | | |
| 5. How happy were you with the level of improvement of balance between the start and finish? (on a scale of 1 to 10) | 7 (2-10) | | | 5 (1-10) | | |
|  | Yes | No | Not sure | Yes | No | Not sure |
| 6. Are you happy with the improvement you have made so far? | 18 | 1 | 9 | 18 | 4 | 7 |
| 7. Do you think the blended Dual Task training programme with PEAK is a good way of achieving the above? | 18 | 1 | 10 | 15 | 5 | 9 |
| 8. Have you noticed any other physical benefits or improvements after completing 3 months of the blended Dual Task training? | 19 | 4 | 6 | 14 | 6 | 9 |
| 9. Were you happy with the way your data is being stored and collected by the PEAK app? | 23 | 1 | 5 | 20 | 2 | 7 |
| 10. Did you receive the support you needed during the programme? | 26 | 0 | 3 | 25 | 1 | 3 |
| 11. How likely will you continue with the training after the study completion? (on a scale of 1 to 10) | 7 (3-10) | | | 6.5 (1-10) | | |
| 12. If you were to continue with the training, how regularly do you think you would do it? |  | | |  | | |
| > three times a week | 1 | | | 1 | | |
| A couple of times a week | 19 | | | 13 | | |
| Just occasionally | 6 | | | 9 | | |
| Whenever I felt like my fitness needed a boost | 3 | | | 3 | | |
| I don't think I would do it again | 0 | | | 2 | | |

Data are presented as median (range).

**Supplementary file 4. Qualitative data with illustrative quotes**

| **Acceptability of the Programme** | | | | | |
| --- | --- | --- | --- | --- | --- |
| ↑ Group Class | ↑ Enjoyed in-class socialising | ↑ Meeting other participants | | | “... I liked talking to the other people in the group...” - DTP26, Focus group 10 |
|  |  | ↑ Being part of a group | | | "Being in a class I think helped tremendously, being all together all doing it as well." - DTP02, Focus group 5 |
|  | ↑ Regular exercise routine | ↑ Liked class format | | | “I think it was set up well and it gave you a good idea what to do at home.” DTP33, Focus Group 8 |
|  |  | ↑ Liked scheduled exercise routine | | | "So it's been good getting that sort of routine when I can and it's been enjoyable to do." - DTP29, Focus Group 6 |
|  | ↑ Valued expert guidance | ↑ In-app support for using app | | | “...How many times did I have to borrow you to, I couldn't even get into the levels, or... And sometimes she'd say, just do that and so I'd try it...” - DTP36, Focus Group 7 |
|  |  | ↑ Direct guidance on exercise | | | “...what we did in the classes, the exercises were fairly well explained, so to do them at home, you didn't have to refer to videos at home...” - DTP03, Focus Group 4 |
| ↕ Usability of PEAK app | ↑ Liked variety of cognitive games | | | | "One of the things I liked about the games is the fact there’s so many of them. So you don’t get bored with one of them." - DTP6, Focus group 6 |
|  | ↑ Liked in-app interactive function | ↑ Real-time feedback | | | “I found them (games) fascinating. Trying to improve, trying to get faster. I got annoyed when I couldn't do something very well. I used to think, come on, I've done pretty well there. And it said, you are better than four percent. And then you do the same game again” - DTP43, Focus Group 7 |
|  |  | ↑ Encouraging feedback | | | “Yes, with the games, it's phenomenal, because if you went up a level, you feel that great sense of satisfaction. Because it would go, awesome... Now you've levelled up, whatever they call it and go up a level, up a level.” – DTP42, Focus Group 7 |
|  | ↕ Device dependent | ↓ Difficult to use on a smaller device | | | “I mean, you're moving around all the time, especially with so many exercises and that might not fit in with tapping the right bit of the screen (of a smart phone).” - DTP29, Focus group 6 |
|  |  | ↑ Easier to use on a tablet | | | “I think an iPad would have been better or even a computer with a game, but then you've got to be able to get to it.” - DTP24, Focus group 9 |
| ↓ Barriers to Home-Based Participation | ↓ Reduced motivation | ↓ Competing priorities | | ↓ Household chores | "I thought I’ve got too much to do. And when I say I’ve got too much to do, the bungalow is quite large really and I’m so slow at doing everything ... and that’s what takes my time to be honest." - DTP14, Focus group 3 |
|  |  |  | ↓ Caregiving | | “But sadly, the reality is you've got grandchildren, you've got holidays, whatever and I know it sounds ridiculous, but I'm afraid that's human nature, isn't it?” - DTP42, Focus group 7 |
|  |  | ↓ Difficulty maintaining exercise routine | | | “Although I’m usually organised in my life, it’s so easy to be distracted by other things when you’re at home to actually fit in the practice at home.” - DTP04, Focus group 2 |
|  | ↓ Worried about being injured | ↓ Lack of confidence exercising alone | | | “My biggest worry is, like with the lunge and stuff like that and I don't do that one, but you're laying up as well, because I'm scared if I'm doing it wrong.” - DTP36, Focus group 7 |
|  | ↓ Lack of space at home | | | | “The thing is, we've got a small house and, in the bedroom, to push things back, push the bed back and it's a large bed, push the bed out of the way to be able to do the exercises, the exercise with the Peak. " - - DTP29, Focus Group 6 |

Symbols indicate positive (↑), negative (↓), and neutral (↕) feedback.

| **Feasibility of implementation of DT programme in NHS Pathways** | | | |
| --- | --- | --- | --- |
| ↓ Difficulty in recruitment | ↓ Majority patient population ineligible | ↓ High frailty level | “...the patients that we get who are referred into secondary care are patients who were probably too low a level to be completing that...” - HCP03, Focus group 1 |
|  |  | ↓ Do not own a smartphone | “Not all of them have access to phones and if they've got a cognitive impairment as well, they're not always going to know how to use their phone... even if they don't have cognitive problems, a lot of them because of the population of their age, they're not really familiar with the technology aspect.” - HCP07, Focus group 2 |
|  |  | ↓ Have progressive neurological conditions | “Because of the risk of people falling. And these are going to be people who are comorbid and have other problems”- HCP11, Interview 1 |
| ↕ Infrastructure readiness | ↓ Lack of space to deliver group class | | “...we don't have people who could deliver this intervention, nor the space to deliver the intervention within general practice.” - HCP11, Interview 1 |
|  | ↑ Feasible in a pre-existing group class model | | “...the balance class would run and fit it in there, whether there's certain aspects of it that could come into our balance, our balance class is definitely the place where it would sit... The ease it would probably be if you could just take it and put it in, it would work.” - HCP 16, interview 4 |
| ↑ Different modes of delivery | ↑ Feasible via existing group-delivery structures in secondary care services | | “So, we perhaps we could look at that because we run our own kind of like balance, rehab exercise groups kind of thing and they have quite good uptake and attendance.” - HCP10, Interview 2 |
|  | ↑ Feasible as group class via NHS community Care Services | | “...it could be delivered in community care and the community setting I think quite readily, but obviously you'd need some interaction between the primary care and the community setting in order to do that.” - HCP11, interview 1 |
|  | ↑ Feasible as one-to-one prescribed therapy in secondary care services | | “Yeah, because for the class we would need an area where to have them and things, but we could show them how this needs to be done when the patient is here. (one-on-one delivery)” - HCP15, Interview 3 |

Symbols indicate positive (↑), negative (↓), and neutral (↕) feedback.

**Supplementary File 5. ModRUM-based unit costs and changes in Health Care Resource Use before and after the DT programme**

| **Resource Use** | Pre | | Post | | Change Cost (£) |
| --- | --- | --- | --- | --- | --- |
|  | Total_cost | Avg_cost | Total_cost | Avg_cost |  |
| A&E Visits | 4368.00 | 132.36 | 2730.00 | 82.73 | -49.64 |
| Face-to-face outpatient appointments | 12276.00 | 372.00 | 11682.00 | 354.00 | -18.00 |
| Telephone outpatient hospital appointment | 455.00 | 13.79 | 490.00 | 14.85 | 1.06 |
| Hospital day case | 10310.00 | 312.42 | 3093.00 | 93.73 | -218.70 |
| Hospital overnight stays | 2376.00 | 72.00 | 1584.00 | 48.00 | -24.00 |
| GP appointments | 2835.00 | 85.91 | 2610.00 | 79.09 | -6.82 |
| GP telephone appointments | 393.20 | 11.92 | 265.41 | 8.04 | -3.87 |
| GP appointment at home | 1340.00 | 40.61 | 268.00 | 8.12 | -32.48 |
| Health or walk-in centre | 12096.00 | 366.55 | 4515.00 | 136.82 | -229.73 |
| Telephone or online with any other healthcare professional | 145.28 | 4.40 | 27.24 | 0.83 | -3.58 |
| Any other healthcare profession at home | 1476.00 | 44.73 | 1230.00 | 37.27 | -7.45 |

**Supplementary File 6. Unit cost of Personal Social Services Research Unit (PSSRU) (2024) for each resource in ModRUM.**

| **Resource Use** | **Unit Cost (£)** | **Cost Source (PSSRU)** |
| --- | --- | --- |
| A&E Visits | 273 | "Emergency care" from Table 6.1.1: Unit costs for hospital services |
| Face-to-face outpatient appts | 198 | "Consultant Led" from Table 6.1.1: Unit costs for hospital services |
| Telephone outpatient hospital appointment | 35 | "Consultations" from Table 7.1.1: Summary of patient resource use and costs following discharge from acute medical units |
| Hospital day case | 1031 | "Day Cases" from Table 6.1.1: Unit costs for hospital services |
| Hospital overnight stays | 792 | "Non-elective inpatient stays (short stays)" from Table 6.1.1: Unit costs for hospital services |
| GP appointments | 45 | "Per surgery consultation lasting 10 minutes" from Table 9.4.2: Unit costs for a GP |
| GP telephone appointments | 9.83 | "Telephone Calls" from Table 9.5.1: Average cost for all initial primary care actions in response to an e-consultation |
| GP appointment at home | 268 | "Per hour of patient contact （With qualification costs）" from Table 9.4.2: Unit costs for a GP |
| Health or walk-in centre | 129 | "Non Consultant Led" from Table 6.1.1: Unit costs for hospital services |
| Telephone or online with any other healthcare professional | 9.08 | "Nurse-led telephone triage" from Table 9.6.1: Costs and unit estimations for a telephone triage |
| Any other healthcare profession at home | 123 | "Intermediate Care Home-Based Services" from Table 6.2.1: National average unit costs for community health services |
