# Supplementary material for: Supervised and Self-Directed Technology-Based Dual-Task Exercise Training Program for Older Adults With a History of Falls: Mixed Methods Feasibility Study
Source: JMIR Aging. 2026 May 18;9:e87577. doi: 10.2196/87577 (PMC13183345; doi:10.2196/87577)
Supplement: Checklist 2 [file aging-v9-e87577-s003.docx]

**TIDieR checklist**

| **TIDieR item** | **Intervention** |
| --- | --- |
| **1.Provide the name or a phrase that describes the intervention.** | Supervised and self-led technology-based dual-task (DT) exercise training programme for older adults with a history of falls. |
| **2.Describe any rationale, theory, or goal of the elements essential to the intervention.** | Dual-task (DT) training, which combines physical and cognitive exercises simultaneously, has been shown to improve walking speed, executive function, balance, and reduced fall risk in older adults [1, 2].  Mobile applications (apps) can offer varied cognitive exercises alongside balance training, creating a comprehensive intervention [3-5]. However, it remains unclear whether older adults with a history of falls, who are at greater risk of recurrence, would be willing or able to engage in unsupervised, technology-supported DT exercise programme and if it can be delivered in clinical or community settings. |
| **3.Materials: Describe any physical or informational materials used in the intervention, including those provided to participants or used in intervention delivery or in training of intervention providers. Provide information on where the materials can be accessed (e.g. online appendix, URL).** | - PEAK app subscription providing access to all the games on the PEAK app - Music Stand to hold the smart phone - Exercise and PEAK handbooks containing instructions on how to perform recommended exercises and PEAK app games at home. - YouTube videos explaining each exercise (https://www.youtube.com/playlist?list=PLmLYc7b_lLKoaypox9foOFQaMqAD36C2u, https://www.youtube.com/playlist?list=PLmLYc7b_lLKr27NaPfGft0NLtzDmnZGlg) - Exercise calendar diary to annotate number of home sessions completed and any additional notes related to exercise progressions. |
| **4.Procedures: Describe each of the procedures, activities, and/or processes used in the intervention, including any enabling or support activities.** | - Supervised Group Exercise Classes (50-minutes): In Phase 1, all participants attended an exercise class (of 5 to 10 participants) once a week for the 12 weeks. Each class is of 50 minutes (5-minute warm-up at the start, then three static exercises, followed by the three dynamic exercises, and finish with a 5-minute cool-down). The static exercises are performed with cognitive games on the PEAK app. - Self-directed, home-based exercise sessions: Participants are asked to perform the same DT exercises at home on another two days in the same week after each class in phase 1, and three times a week during Phase 2, the self-directed, home-bases DT part of the programme. - Educational session: An in-person educational session for falls awareness was conducted for the participants at the end of Phase 1. |
| **5.For each category of intervention provider (e.g. psychologist, nursing assistant), describe their expertise, background and any specific training given.** | - Supervised Group Exercise Classes (50-minutes): These exercise classes are delivered by experienced physiotherapists (TK, HT) from the University Hospitals Birmingham. A researcher (PM), who has completed the Good Clinical Practice course by the National Institute for Health and Care Research, is also always present in the class. - Self-directed, home-based exercise sessions: All participants have the contact details of a researcher (PM) and are instructed to get in touch if they need assistance. - Educational session: This session is delivered by the same qualified physiotherapist who conducted the classes. |
| **6.Describe the modes of delivery (e.g. face-to face or by some other mechanism, such as internet or telephone) of the intervention and whether it was provided individually or in a group.** | - Face-to-face delivery (provided as a group): Supervised group exercise classes and educational session - Self-administered: Self-directed home-based exercise sessions |
| **7.Describe the type(s) of location(s) where the intervention occurred, including any necessary infrastructure or relevant features.** | The supervised group exercise classes were delivered in a community venue and the home-based exercise sessions were conducted by each participant in the homes by themselves. The education session was conducted at the same venue as for the supervised group exercise classes. |
| **8.Describe the number of times the intervention was delivered and over what period of time including the number of sessions, their schedule, and their duration, intensity or dose.** | 24 weeks of Dual Task programme: 1 x 50 minutes per week of group classes and 2 x 50 minutes of home-based sessions per week for weeks 1 to 12, 3 x 50 minutes of home-based sessions per week for weeks 13 to 24. |
| **9.If the intervention was planned to be personalised, titrated or adapted, then describe what, why, when, and how.** | - The physiotherapist leading the class provided individual support and adjustment to the exercises to each participant to ensure that everyone was able to engage with the DT exercise training as per their capacity. - Participants could choose from the 18 games selected from the PEAK app to completed the static exercises based on their preferences. - Participants could perform the home-based sessions at any time at their own convenience. |
| **10.If the intervention was modified during the course of the study, describe the changes (what, why, when, and how).** | To this date, there have been no modifications |
| **11.Planned: If intervention adherence or fidelity was assessed, describe how and by whom, and if any strategies were used to maintain or improve fidelity, describe them.** | - Participants were asked to record the number of home-based sessions completed by them each week in the Exercise Calendar Diary provided to them. - Feedback surveys would be conducted at the end of Phase 1 and again at the end of Phase 2 to provide feedback on the usability, perceived effectiveness and satisfaction from the DT programme based on the feasibility assessment framework [6]. - Focus groups were planned to be conducted with thirty participants to understand their experience participating in the DT programme by a researcher (PM). |
| **12.Actual: If intervention adherence or fidelity was assessed, describe the extent to which the intervention was delivered as planned.** | The intervention was delivered exactly as planned. |

References

1. Rieker JA, Reales JM, Muinos M, Ballesteros S: **The Effects of Combined Cognitive-Physical Interventions on Cognitive Functioning in Healthy Older Adults: A Systematic Review and Multilevel Meta-Analysis**. *Front Hum Neurosci* 2022, **16**:838968.

2. Wang X, Pi Y, Chen P, Liu Y, Wang R, Chan C: **Cognitive motor interference for preventing falls in older adults: a systematic review and meta-analysis of randomised controlled trials**. *Age Ageing* 2015, **44**(2):205-212.

3. Park H, Aul C, DeGutis J, Lo OY, Poole VN, McGlinchey R, Bean JF, Leritz E, Esterman M: **Evidence for a Specific Association Between Sustained Attention and Gait Speed in Middle-to-Older-Aged Adults**. *Front Aging Neurosci* 2021, **13**:703434.

4. Yogev-Seligmann G, Hausdorff JM, Giladi N: **The role of executive function and attention in gait**. *Mov Disord* 2008, **23**(3):329-342; quiz 472.

5. Gale CR, Allerhand M, Sayer AA, Cooper C, Deary IJ: **The dynamic relationship between cognitive function and walking speed: the English Longitudinal Study of Ageing**. *Age (Dordr)* 2014, **36**(4):9682.

6. Boongird C, Keesukphan P, Phiphadthakusolkul S, Rattanasiri S, Thakkinstian A: **Effects of a simple home-based exercise program on fall prevention in older adults: A 12-month primary care setting, randomized controlled trial**. *Geriatr Gerontol Int* 2017, **17**(11):2157-2163
